# Surveillance indicators for potential reduced exposure products (PREPs): developing survey items to measure awareness

**DOI:** 10.1186/1477-7517-6-27

**Published:** 2009-10-19

**Authors:** Karen Bogen, Lois Biener, Catherine A Garrett, Jane Allen, K Michael Cummings, Anne Hartman, Stephen Marcus, Ann McNeill, Richard J O'Connor, Mark Parascandola, Linda Pederson

**Affiliations:** 1 Mathematica Policy Research, Cambridge, MA, USA; 2 Center for Survey Research, University of Massachusetts Boston, Boston, MA, USA; 3 American Legacy Foundation, Washington, DC, USA; 4 Department of Health Behavior, Roswell Park Cancer Institute, Buffalo, NY, USA; 5 National Cancer Institute, National Institutes of Health, Washington, DC, USA; 6 Division of Epidemiology and Public Health, University of Nottingham, Nottingham, UK; 7 Beth-El College of Nursing & Health Sciences, Univ of Colorado, Colorado Springs, CO, USA

## Abstract

**Background:**

Over the past decade, tobacco companies have introduced cigarettes and smokeless tobacco products (known as Potential Reduced Exposure Products, PREPs) with purportedly lower levels of some toxins than conventional cigarettes and smokeless products. It is essential that public health agencies monitor awareness, interest, use, and perceptions of these products so that their impact on population health can be detected at the earliest stages.

**Methods:**

This paper reviews and critiques existing strategies for measuring *awareness *of PREPs from 16 published and unpublished studies. From these measures, we developed new surveillance items and subjected them to two rounds of cognitive testing, a common and accepted method for evaluating questionnaire wording.

**Results:**

Our review suggests that high levels of awareness of PREPs reported in some studies are likely to be inaccurate. Two likely sources of inaccuracy in awareness measures were identified: 1) the tendency of respondents to misclassify "no additive" and "natural" cigarettes as PREPs and 2) the tendency of respondents to mistakenly report awareness as a result of confusion between PREPs brands and similarly named familiar products, for example, Eclipse chewing gum and Accord automobiles.

**Conclusion:**

After evaluating new measures with cognitive interviews, we conclude that as of winter 2006, awareness of reduced exposure products among U.S. smokers was likely to be between 1% and 8%, with the higher estimates for some products occurring in test markets. Recommended measurement strategies for future surveys are presented.

## Background

Over the past decade, tobacco companies have introduced cigarettes and smokeless tobacco products with purportedly lower levels of some toxins than conventional cigarettes and smokeless products. These new products, named by the Institute of Medicine as Potential Reduced Exposure Products (PREPs)[1], have typically been introduced into regional test markets in the U.S. rather than nationwide, which means they are not widely recognized by name or description by most consumers[2,3]. Although some in the public health community welcome the introduction of PREPS, as they may offer a harm reduction opportunity to current tobacco users, other public health advocates do not believe that enough research - particularly long term research - has been done to know whether PREPs, even if proven to have reduced toxins, actually present a reduced health risk to smokers [4]. The concern in the public health community is that tobacco users who might have been motivated to quit may abandon those quit plans if they believe that an alternative, less hazardous option exists with the PREPs. Likewise, former tobacco users could be tempted back to use and non-users could be tempted to initiate use if they too believe that PREPs present lower health risks than conventional tobacco products [5,6]. Having faced a similar situation with the introduction of "light" cigarettes - i.e. new product, insufficient research about its health implications, effective tobacco industry marketing, and subsequent documentation of no public health benefit and possible public health harm - public health officials are cautious about PREPs. Preliminary studies suggest that smokers are interested in trying PREPs, particularly combustible ones, and perceive them to have lower health risks than cigarettes [2,7-9]. On the other hand, despite well-documented evidence that smokeless tobacco is substantially less harmful than cigarettes [10-16], studies demonstrate that most consumers rate them as being as harmful as cigarettes, if not more harmful [17,18]. Thus, public health experts have called for the development of a science to evaluate both the products and the public's response to them, as well as ongoing population surveillance [1,19,20].

This paper describes the development of survey items that could aid in the ongoing surveillance of PREPs in U.S. markets. The first phase in the development effort was a review and critique of current survey measures. The second phase included developing a surveillance instrument and evaluating it by means of two rounds of in-depth cognitive interviews. This paper addresses measurement of product awareness. Other domains, including risk perception, current use, interest in use, and trial, are covered in the full technical reports [21,22]. The decision to focus first on awareness, and not the other domains, is based on the fact that without a valid measure of awareness - one that allows the researcher to be confident that the respondent is thinking of the specific products being studied - measures of risk perceptions, current use, and intentions regarding future use are of little value.

There are three major challenges in developing surveillance measures. The first challenge is the fact that awareness is currently very low. Many PREPs are available only in limited test markets. Another challenge is that the PREPs themselves and their marketing often change without notice, making comparisons in awareness, use and risk perceptions difficult to make over time. A third challenge is the fact that there is no single, agreed-upon definition of a PREP across the public health community. Regardless of these hurdles, it is important to develop survey measures now, to be prepared for the large scale introduction and marketing of PREPs that is coming, and it is vital to have stable measures that can be used across surveys so that comparisons can be made with confidence that observed changes over time and across surveys are due to true changes and not to differences in survey measures.

## Methods

Currently available survey measures, as of 2006, were identified by reviewing published literature on consumer reactions to PREPs (using a number of search terms, including PREPs, new tobacco products, and reduced exposure) and by soliciting input about ongoing and unpublished studies from members of the project advisory group, members of the Tobacco Harm Reduction Network, and other members of the tobacco research community. Additional file 1 is a list of the 16 studies that were identified, seven in the published literature (A1 thru A7) and nine of which were unpublished (A8 through A16). We reviewed all of the PREPs-related questions as well as data on responses to the questions where they were available. A full listing of PREPs-related questions from the included studies is available from the first author. This review of measures and results included analysis of the question features and creation of a taxonomy of question structure. From this we developed new surveillance items using many of the previously identified item features. We subjected the new questions to two rounds of cognitive testing, a common and accepted method for evaluating questionnaire wording [23]. Cognitive testing is done with intensive, one-on-one interviews in which participants are usually asked to summarize their understanding of the meaning of questions and to provide a narrative explanation of how they arrived at an answer to a question by concurrently thinking out loud as they answer the question. This allows an evaluation of the question-and-answer process, question comprehension, how answers were formed, and what the answers mean, towards the goal of making sure that there is consistency of interpretation across respondents [23].

In January 2007, seven cognitive interviews were conducted in the Boston, MA area, where at least one combustible PREP had been available, and one month later, eight interviews were done in Austin, TX, a test market for Camel Snus, a smokeless PREP. The respondents, 11 smokers and four former smokers, were recruited from newspaper advertisements and campus postings. The interviews were conducted by two senior level researchers and one professional interviewer. Respondents were paid $50 for their participation. The interviews, which used concurrent think-aloud techniques, took approximately 45 minutes to complete. All interviews were audio-taped with participants' permission. Variations in wording were tested in these cognitive interviews and probing questions were used to understand respondents' comprehension of the questions. For example, if a respondent said she was aware of a PREP, she would be asked to describe what she knew about it to see if she was really talking about the right product. We probed sufficiently to determine whether or not we believed she answered the tested awareness item correctly and, thus, made a judgment as to whether or not the tested item yielded an accurate or inaccurate indicator of awareness.

## Results and Discussion

The following sections address surveillance of awareness of PREPs and are organized to first summarize current measures, second to critique those measures and use them as a way to develop a new measure of awareness, third to present the results of the cognitive interviewing of a new measure or awareness, and fourth to recommend a surveillance measure. There are measures relating to both combustible and non-combustible PREPs, and while the following summary focuses on combustibles, the results for non-combustibles are presented where they are different or important. When referring to the unpublished studies or survey items, we note their sequence number from Additional file 1.

### Summary of Existing Measures

**Figure 1:**
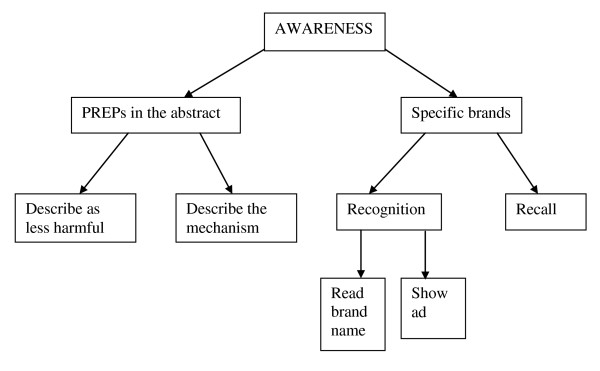
**The Structure of PREPs Awareness Questions**. This figure shows that the initial distinction between question types is that they ask about PREPs in the abstract or they ask about specific PREPs brands. Within each of those groups, there are further divisions, as shown in the figure.

In our review of current measures, we identified the structure of PREPs awareness questions. Figure 1 is a summary of the structure, showing that the initial distinction between questions is whether they ask about PREPs in the abstract or about specific PREPs brands. Within each of those groups, there are further divisions.

As shown on the left side of the figure, a common strategy for measuring awareness of a product is to describe the general concept and ask whether the respondent is aware of such products. There have been two ways of asking about this type of conceptual awareness:

#### Description of PREPs as "less harmful"

Five of the reviewed surveys [18, 24, A8, A10, A15] had a question that asks whether a respondent is aware of a new cigarette described generally as designed to be "less harmful". Items using this strategy yield rather high awareness estimates, with reports of over one-third of smokers having heard of such "less harmful" cigarettes [18, A8, A15].

#### Description of the mechanism for harm reduction

A second way to measure general awareness of PREPs is to start the question with a description of the mechanism by which the harm reduction is supposed to be achieved, such as the following example:

Tobacco companies have recently introduced products that look like cigarettes, but which heat the tobacco, instead of burning it. They claim that these products contain less tar and produce less environmental tobacco smoke. They also say they that they give a lower concentration of cancer-causing chemicals in the smoke. (A9)

When asked if they had heard of these products, only about 13% of smokers said Yes.

As shown on the right side of Figure 1, two general approaches have been used to measure awareness of specific PREPs products: 1) recognition of specific products by either presenting brand names or showing a product advertisement and 2) unaided recall of products.

#### Presenting names of one or more specific PREPs

Use of a recognition question, in which the brand name is provided and respondents indicate whether they have heard of it, often yields high reports of knowledge of specific brands, as high as 53% [25].

#### Show a product advertisement

In two studies [26, A14], respondents were shown advertisements for specific PREPs brands and were then asked if they had seen the ads for these brands before. Among smokers in the one study with available data, awareness measured this way was just below 8% for the PREP shown [26].

#### Unaided recall of brand names

Generally, use of recall strategies, which require respondents to come up with brand names on their own, yields very low reports, and many of the brands that respondents name are not PREPs at all. In two of the studies, none of the brands of "less harmful" tobacco products recalled were PREPs [24, A10]. Some of the named products were so-called "natural" cigarettes (such as American Spirit) or nicotine replacement products (such as Nicorette and Nicoderm). In the other four studies that used unaided recall [18, A8, A9, A15], the reports of actual PREPs brands ranged from about 1% to about 8% of smokers. There were many reports of non-PREPs, including American Spirit and Quest, a nicotine step-down brand.

Table 1 summarizes the current measures of awareness and the estimates they yield.

**Table 1:** Current Measures of Awareness of Combustible PREPs

**HOW SURVEYS ASK ABOUT AWARENESS**	**Percent of smokers affirming awareness**	**Studies with data** (citation numbers shown)*
Describe PREPs as "less harmful"	19-40%	[18]; (A8. A10, A15)

Describe mechanism of PREP	13%	(A9)

Recognition - read names	11-53%	[24,25]; (A11)

Recognition - show a product advertisement	8%	[26]

Unaided recall	0-8%	[18,24]; (A8, A9, A10, A15)

*Citations A8 through A15 refer to unpublished studies listed in Additional file 1.

At the time of this review, only two surveys [25, A13] had attempted to measure awareness of non-combustible PREPs by naming them (Ariva, Revel, Exalt, and Stonewall) along with other types of PREPs and asking the respondent if he/she had heard of them. Among the four smokeless products listed, rates of awareness among smokers ranged from 3% to 6% [25].

### Critique of Existing Measures

The two ways of asking about PREPs awareness in the abstract - describing them as "less harmful" and describing the mechanism - each has limitations. The description of PREPs as less harmful followed by a question asking if they've heard of such a product results in artificially high estimates of awareness because respondents may understand the question to include products marketed as "natural" or "additive free". This is apparent when there is a follow-up question (unaided recall) asking the respondent to name a "less harmful" product, and they name brands such as American Spirit and Winston No Bull, which are not PREPs. It is not surprising that consumers view products such as American Spirit and Winston No Bull as less harmful, since they are marketed as "natural" or "additive-free", messages that are associated with being healthier. There is also a history of consumers' misunderstanding the relative health risk of different tobacco products, including smokeless [18] and light cigarettes [27]. The abstract question about PREPs awareness by describing the mechanism of the PREP is a difficult approach for ongoing surveillance. The difficulty is that the mechanism used in the different products are highly variable and may not be known or understandable when described to the consumer. Further, it is not a good choice for ongoing surveillance because of the variability. A good surveillance item relies on stable measures that can be compared across years and across markets as the new products emerge.

The two ways of asking about awareness of specific PREPs brands - through recognition or recall of brand names - also have limitations. As shown in summary Table 1, the rates of awareness resulting from recognition measures are often very high, ranging from 11% of smokers (A11) to 53% of smokers [25]. We suspected that these rates were over-reports because a number of PREPs brands share names with other products. For example, Eclipse, Accord and Omni are all combustible PREPS that share their name with, respectively, a chewing gum, a car, and a hotel. When respondents say they recognize those names, we cannot be certain that they are thinking of the cigarette brand or if they simply recognize the name more generally and, therefore, report having heard of it. An alternative recognition approach, showing a product advertisement, has the same limitation as describing the mechanism, which is that it is an impractical approach for ongoing surveillance.

Use of an unaided recall awareness question typically follows a question that asks about PREPs in the abstract. As described earlier, we know that what respondents name in these unaided recall questions is often not a PREP at all, but other products that they consider less harmful, such as products advertised as more natural, additive free, or quit aids.

We used these critiques of existing measures to develop a new awareness measure to test in cognitive interviews.

For the two rounds of cognitive interviews, we asked first about conceptual awareness of products described as less harmful, followed by unaided recall to identify over-reports in the conceptual awareness question, and then recognition items, including fictitious items to gauge false reports of similar sounding names. Following are the items used to measure awareness of combustible PREPs:

Conceptual awareness

New types of cigarettes are now being developed that are supposed to be less harmful than ordinary cigarettes. Have you heard of such products?

Unaided recall

(If yes to conceptual awareness) Can you recall any brand names of these products?

(If yes to above) Please tell me the names of any you recall.

Follow-on recognition task

I'm going to read you the names of some (other) relatively new cigarettes. For each one, please tell me whether or not you have ever heard of it.

### Cognitive Interview Results

In cognitive interviews, six of 15 participants said Yes to the conceptual awareness question about combustible PREPs. Of those, only two recalled the name of legitimate PREPs. Cognitive probes (e.g., What do you know about them? What have you heard about them?) yielded appropriate descriptions of the brands from the two respondents that confirmed their legitimate recollection. A third person who said Yes to the conceptual awareness question could not recall any brand names but described PREPs in a way that suggested that she really was aware of them (i.e. she reported that they were lower in carcinogens and that she had seen a TV documentary about them). The three other participants who said Yes to the combustible PREPs awareness question went on to name non-PREPs like American Spirit, Winston No Bull, nicotine products, and even Newport Lights.

The tradeoff, then, is between a small under-estimate of awareness if a respondent is required to name a PREP in order to be considered aware (one of the three who was truly aware of PREPs was not counted because she failed to name a PREP) or an even larger over-estimate if only the yes/no conceptual awareness question is used to estimate awareness (six who said they were aware instead of the three who said they were aware and really were).

False recognition reports were common among participants. We included distracter brand names - two out of seven brands listed in the Boston interviews and three out of eight brands listed in Austin. Ten of the 15 participants interviewed recognized 12 of the listed brands, but half of those mentions were for our distracter products - three said they "recognized" Kool Silver and three "recognized" Westin. As a result of this high level of false recognition, we are doubtful of the reports of actual PREPs because two mentions were for Eclipse and two were for Marlboro Ultrasmooth, both of which could be confused with other products and be "recognized" by virtue of their familiar-sounding names. Probes into what was known about these brands (legitimate and bogus) yielded no convincing information that would lead us to believe that the respondent was truly aware of the PREPs.

Considering only the recognition responses (ignoring recall for the moment, even though recognition would be different if it did not follow recall), we would conclude that 4 out of 15 participants were aware of combustible PREPs, based only on those who said they recognized a real PREP and in probing, convinced us that they truly were aware. Having the distracter names on the lists works as a benchmark as to how much name recognition is attributable to a familiar name, and not the specific PREP.

Since the new smokeless PREPs were not all being advertised as being potentially less harmful, the introductory question required a somewhat different approach to maximize the likelihood that someone who was aware of any of the products would understand what was being referred to. The unaided recall and follow-up recognition questions were the same as was used for the combustible PREPs.

Conceptual smokeless PREPs awareness

New types of smokeless tobacco products are now available that are put in the mouth but don't involve chewing or spitting. Some come in teabag-like pouches and some come in the form of a lozenge or tablet. Have you heard of any products like this?

Since Austin is a test market for one of the new smokeless PREPs, the results there were very different from results in Boston, where awareness was minimal. Essentially no one in the Boston study group was aware of any smokeless PREPs based on the conceptual description and unaided recall. With regard to recognition, two participants thought they had each heard of one of the named products, but there is no way to know whether these recognitions were legitimate or whether they were simply recognitions of a familiar brand name.

In Austin, however, all eight respondents said "yes" to the conceptual awareness question. Five of these participants named either Camel Snus or a Skoal product in the followup unaided recall question. If we accept as awareness "yes" to the conceptual awareness questions followed by a correct brand name recalled, we would conclude that five of the eight participants in Austin were aware of smokeless PREPs. The actual estimate for Austin should be six of eight respondents, because one individual could not name a brand but clearly described the Stonewall product. Hence, this strategy, as with the comparable combustible measure, slightly underestimated awareness in a test market.

### Recommendation for Measuring Awareness of PREPs

We recommend that awareness of PREPS, both combustible and non-combustible, be measured using the conceptual awareness question, followed by unaided recall of a brand so that those who name non-PREPs can be identified as unaware. These are the items that would be used at present to estimate PREPs awareness. We also recommend asking the recognition series, including distracter names, as a way to monitor changes in both real and likely false recognition of PREPs. However, we do **not **recommend using the recognition data, alone or in conjunction with the other data, to **estimate **current awareness of PREPs at this time. As awareness increases, and the false reports drop to a small proportion of recognized brands, it will be time to consider how to combine the recall and recognition data to estimate awareness. Additional file 2 shows the recommended series of items to measure awareness of PREPs. It is important to consider that this recommended strategy for measuring awareness is feasible for interviewer-administered or web surveys where skips are possible and open-ended responses are feasible. This strategy is not feasible for self-administered surveys.

### Unresolved Issue - Definition of a PREP

In order to monitor awareness of PREPs, researchers need some agreement about what constitutes a PREP. Although the definition originated by the Institute of Medicine includes pharmaceutical agents, such as nicotine replacement products [1], they were not the focus of our study and, thus, were excluded from our definition. Likewise, our definition excludes quit aids, such as Quest, and is modeled after the one provided in Hatsukami and Hecht, focusing on "tobacco products that have been modified or designed in some way to reduce users' exposure to tobacco toxins" [19]. Arguably, the essential characteristic of most definitions is that they are talking about products designed to yield reduced exposure to toxins. The problem is that many of these products are not currently being advertised as entailing reduced exposure. To an important extent, the design of the surveillance items being recommended in this report is determined by current PREPs marketing practices. Specifically, because the new smokeless products are not being advertised as less harmful than cigarettes, the recommended awareness questions do not include a statement that they are "supposed to be" or "claim to be" less harmful. Most of the combustible PREPs for which awareness is being assessed were advertised as less harmful during the time that they were being actively marketed, so the awareness question recommended here includes language about reduced harm. However, marketing strategies are likely to change. Philip Morris did not make any health claims while test-marketing Marlboro Ultrasmooth, and the Eclipse and Advance web sites, which originally featured extensive health claims, are no longer readily accessible. This issue complicates assessment of awareness of PREPs because although the public health community may be aware that the product design suggests an effort to reduce exposure to particular toxins, the general public may be unaware of that effort while still being aware of the new product.

### Surveillance in other important areas

This paper covers only one of the domains of surveillance, but there are a number of others that are important for ongoing consideration. Specifically, interest in use, risk perception and trial will be important areas to monitor as PREPs become more common. Once one is reasonably confident of having an accurate indicator of product awareness, it will be important to have good measures in these areas and to know whether consumers are over-estimating or under-estimating the risks or potential health gains of various products. This will be particularly important as marketing messages change.

Because all indications are that population levels of awareness of PREPs are quite low, national population monitoring of patterns of current use and impact on changes in other tobacco use behavior seems unnecessary at this time. As levels of PREPs awareness and trial increase, as is likely to be the case with the new smokeless products being introduced by the major cigarette manufacturers, we would advocate introduction of current use measures in the surveillance program. Given the long lead time required to introduce questions on Federal-level surveys, it is, perhaps, appropriate to consider introducing those questions now, so as to be prepared for the increase in use when it happens. Likewise, communications research that examines advertising, packaging, and health claims for specific products are critical and should be conducted. In order to be prepared to respond should these products become more widely available, it is important to learn how best to construct public health messages and policy to both maximize potential public health benefits of less harmful tobacco products and minimize negative health consequences.

In addition to the PREPs covered in this report, there are a variety of other products that may be believed to be less harmful than cigarettes, and which may be used by consumers in an effort to reduce their tobacco-related health risks. These include conventional smokeless tobacco, medicinal nicotine, e-cigarettes, other non-tobacco nicotine products, and tobacco products that claim to be "natural" (i.e. have no additives), etc. A thorough understanding of population perceptions about ways to reduce the risks of smoking (aside from quitting), along with the prevalence of behaviors believed by consumers to reduce risk, would be a useful endeavor.

It is also important to recognize that population surveillance is just one way to collect data about PREPs. In the development of a science to evaluate PREPs, other strategies, including lab research, qualitative studies, and review of tobacco industry information, are needed to get a full picture. This paper focuses on population surveillance measures from surveys and does not cover the full array of monitoring strategies. However, surveys are particularly useful for estimating prevalence of awareness, interest and use, as well as perceptions, and it is important to develop accurate surveillance measures to track changes in these areas over time, as the number of PREPs on the market increases.

## Conclusion

A review of existing research on population awareness of PREPs as well as results of cognitive interviews suggest that awareness of PREPs is quite low at this time, except in active test-markets, and is lower than some published studies suggest. In order to monitor changes in awareness, we propose using a set of questions that appear to result in reasonably accurate estimates of awareness of a particular group of products; the proposed items are presented in Additional file 2.

Given the currently very low levels of awareness and the decision of the tobacco companies to introduce most of their new products into test markets, it may fall on states to use their rapid surveillance systems to take the lead in measuring awareness. States in which there are regional test markets could add questions to the Behavioral Risk Factor Surveillance System (BRFSS) and the Adult Tobacco Survey (ATS) to start the PREPs surveillance process. At this stage, state surveys would be more successful than national studies that will not have enough respondents within test market regions to detect small movements in awareness. These states could compare test market areas to non-test market areas within their states, as well as make comparisons across states, if surveys adopt the same wording. Once awareness increases, it will be time for surveys to include surveillance measures in other important domains. However, Camel Snus, a smokeless PREP, was released nationally in 2009 [28], so it is certainly the right time to consider adding an awareness surveillance measure at the national level as well.

It must be acknowledged that the evaluation of items in this paper was limited by the low number of respondents and by the fact that those included were of relatively high socio-economic status. Prior to any large scale use of these survey items, we recommend further pre-tests be done using other survey modes (telephone and self-administered), and that an effort be made to include respondents with lower levels of education and with greater cultural diversity.

## Competing interests

The authors declare that they have no competing interests.

## Authors' contributions

KB and LB worked on the review of previous studies, designed the research project, completed the cognitive interviews of draft questions, and worked on the two reports on which this manuscript is based. KB drafted this manuscript; LB contributed extensively to its completion.

CAG provided all manner of research support on the earlier reports for this project, including literature reviews, and on this manuscript. SM and MP were the research officers on the original project from which this manuscript derives. AM, RO, MP and LP were on the project advisory group, which helped design the interview questions and to interpret the results. All co-authors participated in discussions of the findings, provided numerous reviews of this manuscript, and read and approved the manuscript.

## Supplementary Material

Additional file 1 **Studies included in the summary**. This is a list of the published and unpublished studies that were included in this study summary. They represent the currently available survey measures as of 2006. Click here for file

Additional file 2 **Recommended items**. This is a list of the recommended series of items to measure awareness of PREPs. Click here for file
