# Supplementary material for: Surveillance indicators for potential reduced exposure products (PREPs): developing survey items to measure awareness
Source: Harm Reduct J. 2009 Oct 19;6:27. doi: 10.1186/1477-7517-6-27 (PMC2770517; doi:10.1186/1477-7517-6-27)
Supplement: Additional file 1 — Studies included in the summary. This is a list of the published and unpublished studies that were included in this study summary. They represent the currently available survey measures as of 2006. [file 1477-7517-6-27-S1.DOC]

**Additional file 1: Studies included in the summary**

| **Published Studies** | | |
| --- | --- | --- |
| **First Author** | **Citation** | **Universe**  **Sample Size** |
| (A1) Hamilton WL | Hamilton WL, Norton GD, Ouellette TK, Rhodes WM, Kling R, Connolly GN. Smokers' responses to advertisements for regular and light cigarettes and potential reduced-exposure tobacco products. *Nicotine & Tobacco Research.* December 2004; 6 (Supplement 3):353-362. | Convenience sample of Massachusetts smokers 18 – 65 years  n=599 |
| (A2) Hughes JR | Hughes JR, Keely JP, Callas PW. Ever users versus never users of a "less risky" cigarette. Psychology of Addictive Behaviors. 2005;19(4):439-442. | Convenience sample of smokers  n=287 |
| (A3) Hund LM | Hund LM, Farrelly MC, Allen JC, et al. Findings and implications from a national study on potential reduced exposure products (PREPs). Nicotine & Tobacco Research. 2006;8(6):791-797 | Adults 18+ years  n=6977 (n=1174 smokers) |
| (A4) O’Connor RJ | O'Connor RJ, Hyland A, Giovino GA, Fong GT, Cummings KM. Smoker Awareness of and Beliefs About Supposedly Less-Harmful Tobacco Products. *American Journal of Preventive Medicine.* 2005;29(2):85-90. | Smokers 18+ years  n=2028– wave 2 |
| (A5) Parascandola M | Parascandola M, Augustson E, O'Connell M. Consumer Awareness and Attitudes Related to Characteristics of PREP Use In a National Population Sample. Poster presented at: NCTOH in Minneapolis, MN; 2007. | Current and recent (past 5 year) former smokers  n=44,946 (n=34,644 smokers) |
| (A6) Parascandola M | Parascandola M, Hurd A, Augustson E. Consumer Awareness and Attitudes Related to New Potential Reduced-Exposure Tobacco Products. *Am J Health Behav.* 2008;32(4):431-437. | Convenience sample of adults  n=9736 (n=2044 smokers) |
| (A7) Shiffman S | Shiffman S, Pillitteri JL, Burton SL, Di Marino ME. Smoker and ex-smoker reactions to cigarettes claiming reduced risk. Tobacco Control. 2004;13:78-84. | Convenience sample of smokers and former smokers  n=1499 (n=1000 smokers) |

| **Unpublished Studies** | | |
| --- | --- | --- |
| **Author/Contact** | **Study Name** | **Universe**  **Sample Size** |
| (A8) Gary Giovino, Ph.D.  Professor and Acting Chair, Department of Health Behavior, School of Public Health and Health Professions, University at Buffalo, The State University of New York  Personal communication (written)  6/2006 | Assessing the Hard Core Smoker | Smokers 25+ years  n=1000 |
| (A9) Michael Cummings, Ph.D., MPH  Chair, Department of Health Behavior  Division of Cancer Prevention and Population Sciences Roswell Park Cancer Institute  Buffalo New York  Andy Hyland, Ph.D.  Research Scientist, Department of Health Behavior Division of Cancer Prevention & Population Sciences Roswell Park Cancer Institute  Buffalo New York  Personal communication (written) 5/2006 | Beliefs About Nicotine Delivery Devices | Smokers 18+ years  approx n=1041 |
| (A10) Murray Kaiserman  Director, Research Evaluation and Surveillance, Tobacco Control Programme, Healthy Environments and Consumer Safety Branch (HECSB)  Health Canada  Personal communication (written) 5/2006 | Consumer Interest in Potential Reduced Exposure Products: Results of a National Survey | Canadian Smokers  n=537 |
| (A11) Lois Biener, Ph.D.  Senior Research Fellow  Center for Survey Research  Univ. of Massachusetts at Boston  Boston, MA  Karen Bogen, Ph.D.  Senior Research Fellow  Center for Survey Research  Univ. of Massachusetts at Boston  Boston, MA | Consumer Perception of PREPs Survey | Convenience sample of current and former smokers in Mass. Towns with high rates of smoking  n=200 (n=177 smokers) |
| (A12) Mark Parascandola, Ph.D., MPH, Cancer Prevention Fellow, National Cancer Institute, Bethesda, MD  Personal communication (written) 8/2006 | Health Information National Trends Survey | Adults 18+ years  n=6369 (n=1246 smokers) |
| (A13) Mark Parascandola, Ph.D., MPH, Cancer Prevention Fellow, National Cancer Institute, Bethesda, MD  Personal communication (written) 8/2006 | Health Information National Trends Survey II | Adults 18+ years  n=5586 (n= 1015 smokers) |
| (A14) Richard O’Connor, Ph.D.  Department of Health Behavior, Division of Cancer Prevention and Population Sciences, Roswell Park Cancer Institute  Personal communication (written)  5/2006 | Marlboro Ultra Smooth Advertising Survey | Convenience sample of New York smokers  n=34 |
| (A15) Lois Biener, Ph.D.  Senior Research Fellow  Center for Survey Research  Univ. of Massachusetts at Boston  Boston, MA | UMass Tobacco Study | Adults 22+ years  n=2140 – wave 3 (n=1080 smokers) |
| (A16) Gary Giovino, Ph.D.  Professor and Acting Chair, Department of Health Behavior, School of Public Health and Health Professions, University at Buffalo, The State University of New York  Personal communication with Dr. Giovino (written)  6/2006  Dianne Barker, M.H.S.  Barker Bi-Coastal Health Consultants Calabasas, CA | Youth Smoking Cessation Survey | Youth smokers  n=2582 - baseline |
