# Supplementary material for: Surveillance indicators for potential reduced exposure products (PREPs): developing survey items to measure awareness
Source: Harm Reduct J. 2009 Oct 19;6:27. doi: 10.1186/1477-7517-6-27 (PMC2770517; doi:10.1186/1477-7517-6-27)
Supplement: Additional file 2 — Recommended items. This is a list of the recommended series of items to measure awareness of PREPs. [file 1477-7517-6-27-S2.DOC]

**Additional file 2: Recommended Items**

**Combustible PREPs**

**1. New types of cigarettes are now available that are supposed to be less harmful than ordinary cigarettes. Have you heard of such products?**

[ ] YES

[ ] NO (SKIP TO 2)

**1a. Can you recall any brand names of these products?**

[ ] YES

[ ] NO (SKIP TO 2)

**1b. Please tell me the names of any you recall.**

____________________ ___________________

____________________ ___________________

**2. I’m going to read you the names of some (other) relatively new cigarettes. For each**

**one, please tell me whether or not you have ever heard of it.**

(EXCLUDE NAMES ALREADY GIVEN IN 1b)

The list of named brands will change with the market. We recommend including several distracter (D) items that do not exist. We used the following list: Eclipse, Accord, Westin (D), Advance, Marlboro Ultrasmooth, Kool Silver (D), Quest, Excite (D).

**SMOKELESS**

**3. New types of smokeless tobacco products are now available that are put in the mouth but don’t involve chewing or spitting. Some come in teabag-like pouches and some come in the form of a lozenge or tablet. Have you heard of any products like this?**

[ ] YES

[ ] NO (SKIP TO 4)

**3a. Can you recall any brand names of these products?**

[ ] YES

[ ] NO (SKIP TO 4)

**3b. Please tell me the names of any you recall.**

____________________ ___________________

____________________ ___________________

**4. I’m going to read you the names of some (other) of these new types of smokeless tobacco products. For each one, please tell me whether or not you have ever heard of it.**  (EXCLUDE NAMES ALREADY GIVEN IN 3b.)

The list of named brands will change with the market. We recommend including several distracter (D) items that do not exist. We used the following list: Ariva, Revel, Exalt, Assert (D), Stonewall, Camel Snus, Newport Bold (D), Taboka, Skoal Dry
